# Terrorism and Mental Health

**Published:** 2004

**Authors:** J. K. Trivedi

**Affiliations:** * Prof & Head, Dept. of Psychiatry, Kings George Medical College, Lucknow. jktrivedi@hotmail.com


*Indian Journal of Psychiatry, 2004, 46(I)7-14*

### Terrorism and Mental Health

**J.K. Trivedi***

Respected Chairpersons and members of the Indian Psychiatry Society.

It is indeed a great honour delivering the presidential address here at the "56th" annual conference of our esteemed society in the city of palaces, Mysore. I am grateful to the honorable members of the IPS who have reposed their faith in me and provided me an opportunity to work for the highest office for a year. I feel overwhelmed whenever I think about the day when I joined psychiatry as my career and since then each day has added depth and meaning to my life. I take this opportunity to convey gratitude for whatever little I am, to the blessings and guidance of my teacher Late Prof. B.B.Sethi, an eminent psychiatrist, a great academician, a visionary and founder of our department at Lucknow. In more than two decades of my professional career as a clinician and as a teacher, many illustrious psychiatrists and stalwarts have inspired and guided me. Whenever I think of those distinguished figures I feel inundated and ponder if I could imbibe even a fraction of their virtues. I owe greatly to all of them.

I felt on crossroads while choosing the topic for presidential address. It was indeed very alluring to speak on one of my areas of research. However " considering the current socio-political scenario, which is effected frequently by terrorism. We routinely see, hear and read about terrorism and its consequences through media. We as the mental health service providers would have an important role to play in training, advising and assisting 'front-line responders 'as well as helping in the management of those with psychiatric and psychosocial problems. It also provides us a unique opportunity and challenge to sensitize the general population to vulnerability of all people to mental disorders and the scope for recovery and healing. I would like to address some of the major issues in relation to a terrorist attack, including its likely psychological effects and the possible intervention strategies to mitigate such effects

### Introduction

Our nation has been the victim of terrorism since long. Almost all the regions of our country including north, south, east and west have been affected by the menace called 'terrorism' on different occasions. In few areas like Northeast and now Jammu and Kashmir it has almost become a perpetual problem. In the post colonial era one of the first insurrections was encountered in Telangana led by the communist. The state of Punjab was torn apart by savage sectarian violence, possibly one of the most brutal since independence. The ongoing insurgency in Jammu and Kashmir, since more than a decade, has resulted in widespread violence and has caused much of the suffering and psychological sequelae as its aftermath. World wide also, several regions are affected by terrorism like, Northern Ireland, Israel, Sri Lanka etc. After the September 11 the issue of terrorism came in the forefront as it affected the most powerful nation of the present times.

Terrorism is a kind of psychological warfare. Historically terror has proved to be an effective instrument of coercion and intimidation of state organizations by various terrorist associations such as the al Qa'ida, Irish republican army, Jaish-e-mohammad, L.T.T.E etc. The mechanism of action to terrorize the society may be different but their purpose remains the same. The mechanisms could be in the form of blasts, suicide terrorism, bio-terrorism, narco-terrorism and financial terrorism.

Different governments and organizations have defined the word "Terrorism" differently. These may slightly differ in their language but the basics remain the same.

Our own Prevention of Terrorism Act (Pota, 2002) has a lengthy definition emphasizing especially upon the very purpose and impact of the act of terrorism by saying "terrorist act produces a prolonged psychological effect on society, disrupts even tempo and tranquility and produces a sense of insecurity in the minds of a section of the society or the whole society ".

Terrorism is defined by Title 22 of the U.S Code(2002) as, "politically motivated violence perpetrated against non-combatant targets by sub national groups or clandestine agents, usually intended to influence an audience".

Definition of terrorism mentioned anywhere has three key criteria, which distinguish terrorism from other forms of violence.

*Prof & Head, Dept. of Psychiatry, Kings George Medical College, Lucknow. email : jktrivedi@hotmail.com

Presidential Address delivered at 56th Annual Conference of Indian Psychiatric Society, held at Mysore 8th to 11th Jan. 2004

1. The act is politically motivated. Terrorism is directed towards goals that are political, that is terrorist actions are intended to guide or influence governmental policies. This criterion emphasizes that the social & psychological antecedents of personally or criminally motivated violence are different than the antecedents of terrorist violence.
2. Secondly, terrorist violence is directed primarily at non-combatants who are not members of the military services, and are part of civilian populations, group or nationalities and not prepared to defend against violence.
3. The third criterion is that sub national groups or clandestine agents commit terrorist attacks. The crux of this criterion is the clandestine nature of terrorism. In situations of declared war or announced conflict such as our three wars with Pakistan, bloodshed and hostilities were expected by the citizens. On the other hand, most of the times victims of terrorism cannot anticipate the attack because of this clandestine feature, making terrorism so unpredictable and alarming.

Literature on psychology of terrorism emphasizes that terrorism is intended to create an extremely fearful state of mind. Moreover, this fearful state is not intended for the terrorist's victim rather it is intended for an audience who may have no relationship with the victims (Kaplan, 1981). Thus aim of the terrorists is to create crippling fear and psychological debilitation in an audience beyond the immediate victim (Jones and Fong, 1994). The eventual purpose of terrorism could be creating mass anxiety, fear and panic; creating helplessness, hopelessness and demoralization; destroying the assumptions about personal security; disruption of the infrastructure of a society and demonstrating the impotence of the authorities to protect the ordinary citizen and their environment. The governments are also likely to use the legal or moral perspective to interpret terrorism. A 'freedom struggle' for some may be 'terrorism' for others. Under certain circumstances, what becomes good may be bad under other conditions. So nothing is absolute.

### Psychology of Terrorism

Different authors have tried to interpret the psyche of the terrorists and the psychology behind the terrorism. While going through all the interpretations I was just thinking what compels us to explain this phenomenon and to what extent we are doing it rightly. Is it the perceived pressure from the others or just our own self-imposed right to over intellectualize all kinds of human behavioural components? Things that however is sure that no single theoretical framework can suffice to explain this complex phenomenon (Kapoor R.L., 1994). There are those like Crenshaw (1990) who sees terrorism as a conscious, intentional decision of a group, reached deliberately. Others like Post (1990) believed in the psychodynamic aspect and opined that terrorist has suffered narcissistic wounds in childhood, which implies that the self is not able to integrate its own good and bad parts. The individual projects on to others the bad parts, the hated and devalued aspects of his psyche. Sprinzak (1990) viewed this occurrence in light of the underlying group dynamics rather than the psychology of individual. According to him there could be three stages of the development of terrorist dynamics. First there is a crisis of confidence with the established political system. Next the legitimacy of system is questioned. It is not just the leaders who are seen to be manipulative but the system itself. Finally the individuals and the society identified with the existing system get depersonalized and dehumanized. Dehumanization allows one to commit atrocities. Joining the militant organization gives relief to an isolated individual, who may become more likely to agree to take part in atrocities.

The Oslo Congress in July 2003 concluded on the following possible factors leading to the emergence of terrorism in a society, which mainly are socio-political in nature:

a. Lack of democracy, civil liberties and the rule of law is a precondition for many forms of domestic terrorism.

b. Failed or weak states lack the capacity or will to exercise territorial control and maintain a monopoly of violence.

c. Rapid modernization correlate strongly with the emergence of ideological terrorism.

d. Extreme ideologies of a secular or religious nature are at least an intermediate cause of terrorism.

e. Historical antecedents of political violence, civil wars, revolutions, dictatorships or occupation may lower the threshold for acceptance of political violence and terrorism.

f. Hegemony and inequality of powers.

g. Illegitimate or corrupt governments h. Powerful external factors upholding illegitimate governments i. Repression by foreign occupation or colonial powers j. The experience of discrimination on the basis of ethnic or religious origin k. Failure or unwillingness by the state to integrate dissident groups or emerging social classes l. The experience of social injustice m. Triggering events like outrageous act committed by the enemy, lost wars, massacres, contested elections, police brutality, that call for revenge or action.

### Psychological sequelae

Amassing of severe or numerous life events in any form can act as strong stressor and affect the mental equilibrium, producing maladaptive patterns of behaviour. Psychological responses to terrorism are a mixture of reactions towards the trauma and also towards a constant fear of being a victim to a traumatic event in the future. Such reaction may vary among individuals depending upon the extent of personal damage in any form, proximity to the place where the act has been committed, brutality of the event, his or her own coping styles, likely expectation of a future repetition and the chronicity of the threat scenario. Despite adversities, in places like Afghanistan, Israel, and Jammu and Kashmir where the populations lie under a constant threat, by and large people survive and enjoy the niceties of the life. Have they become apathetic and insensitive to it? Or they have become resistant to it? Or have accepted the terrorism as a part of their life? The probable answer lies in the remarkable ability of human adaptive behavior which many times protect the affected individuals from being overwhelmed by the lasting threat.

Understanding of the psychological aftermath of terrorism is increasingly acquiring importance as its global threat is intensifying in its extent and frequency. Observations following natural and human induced major trauma described a miscellany of individual reactions. The general effects of threat of terrorism on attitudes, cognitive processing and behaviour have been well documented by psychiatrists, psychologists and social and political scientists. Tyhurst (1951) suggested that, following a major trauma, there is likely to be a triphasic response .In the initial 'impact', survivors will be preoccupied with their present situation and most will be stunned and numbed. During the 'recoil' phase, survivors will want to talk to others and seek support. The reality of what has occurred becomes irresistibly obvious to survivors at the 'post-trauma' phase. During this phase survivors are likely to display a number of emotional reactions, including depression, anxiety and anger (particularly if they consider that their legitimate needs have not been met). Levine and Campbell (1972) and Struch and Schwartz (1989) opined that threat leads to an increased ethnocentrism and anxiety for strangers. Doty (1991) and Marcus et al. (1995) believed that threat promotes intolerance and a willingness to forego basic civil liberties. It also leads to close mindedness and rejection of challenging beliefs (Lodge and Taber, 2000; Rokeach, 1960). Along with "survivor guilt" that is feeling guilty that they are alive when their near and dear ones are dead, many victims think that they should have died in the disaster along with the dead relatives (Myers 1994, Kar, 2000). There is reduced efficiency of memory process (Blaney, 1986) and promotion of both threat related thought content (Gilligan and Bower, 1984) and perceptual hypersensitivity to information concerning threat (Mathews and Macleod, 1986). Liberman and Chaiken (1993) observed that threat biases cognitive processing. Tendency of taking risks also increase under threatening situations (Kahneman and Tversky, 1979). Most of the above findings suggest some degree of cognitive shutdown and biased cognitive processing. With such state of psyche they feel good factor of the life is lost. Considering the WHO definition of "health", which incorporates the sense of well being as an essential component it reflects the ' unhealthy' state of such affected individuals.

Psychological responses of individuals may differ depending upon the perceived personal v/s national or collective threat. Personal threats, especially threats that pose a physical (i.e. job, finances, family and health) danger, are likely to be very affectively arousing and elicit fear to a greater degree than more remote threats to the nation. A national study on reactions to the terrorist attacks on New York and Washington revealed that personal threat was much more likely than national threat to elicit fear, anxiety and related somatic symptoms such as depression and insomnia (Huddy et al.,2002). Research has shown that any form of personal threat and fear leads to a change in personal behaviour designed to minimize exposure to risk (Green berg et al., 1992; Jacobson and Bar-Tal, 1995), also referred as 'constrained behaviour' (Ferraro, 1996). Individuals spend more time with their families, change their plans to travel and use public transportation less frequently. From this perspective, it may be irrational to avoid travelling by train, given the very small percentage of people who die in train accidents, but it is emotionally sensible to avoid travelling if it prevents the arousal of intensely fearful emotions.

### Psychiatric morbidity as it's aftermath

Psychological trauma not only leads to disturbance in the mental equilibrium causing maladaptive behaviour but also results in diagnosable psychiatric disorders. Its recognition has waxed and waned through out the past century. Specific categories such as 'combat neurosis' (Grinker and Spiegel, 1945), 'operational fatigue' and 'shellshock' paved the way for recognition of a general category of post traumatic stress disorder (PTSD), in DSM III (1980) introduced after the description of 'post Vietnam war syndrome' (Figley, 1978). PTSD, though commonly encountered, is not the only form of the psychiatric morbidity in the aftermath of terrorism.

A large number of individuals report medically unexplained physical symptoms (Engel, 2002). Widespread report of chest pain and respiratory problems following the events of September11 were referred as 'World Trade Center syndrome'. Table-1 lists the different forms of psychiatric morbidity associated with terrorism.

**Table-1**

**PSYCHIATRIC MORBIDITY**

- Acute reaction to stress
- Adjustment disorder:
  - Brief depressive reaction
  - Prolonged depressive reaction
  - Mixed anxiety/ depressive reaction
  - Mixed with irritability/anger
- Anxiety states:
  - Generalised anxiety disorder
  - Mixed anxiety and depressive state
  - Panic attacks
  - Dissociative disorder
- Depression
- Post Traumatic Stress Disorder
- Others
  - Exacerbation of pre-existing mental illness
  - Exacerbation of personality traits
  - Neuropsychiatric effects of concussion, head injury, brain damage, epilepsy
  - Alcohol and other substance abuse
  - Enduring personality change

Several epidemiological studies have reported different prevalence rates of psychological disorders among the population, directly or indirectly affected by different forms of violence including terrorism. Majority of the studies have been done in the West. There is a dearth of psychiatric data about PTSD and/or clinical depression following terrorist attacks in the Indian context, which needs urgent attention by Indian researchers (Singh and Singh, 2003).

In a survey done by Galea et al. (2002), among 1008 adults interviewed 5 to 8 weeks after September 11 attack, 7.5 percent reported symptoms consistent with a diagnosis of current PTSD related to the attacks, and 9.7 percent reported symptoms consistent with current depression (with " current" defined as occurring within the previous 30 days). Among respondents who lived south of Canal Street (i.e., near the World Trade Center), the prevalence of PTSD was 20.0 percent. Predictors of PTSD were Hispanic ethnicity, two or more prior stressors, a panic attack during or shortly after the event, residence in south of Canal Street and loss of possessions due to the event. Predictors of depression were Hispanic ethnicity, two or more prior stressors, a panic attack, a low level of social support, the death of a friend or relative during the attacks and loss of a job due to the attack. They found bivariate associations between female gender and both PTSD and depression, a finding that is consistent with the results of most studies (Goenjian et al, 2001;Shore et al 1989). This survey suggested that the prevalence of current PTSD and current depression were approximately twice the baseline respective values of 3.6% (with in previous year) and 4.9% (with in previous 30 days) in the U.S. population (NIMH, 1999; Blazer et al., 1994).

In another national survey (Schuster et al., 2001) 560 U.S. adults were interviewed about their reactions to terrorist attacks and their perceptions of their children's reactions, 3 to 5 days after September 11. Forty-four percent of the adults reported one or more substantial symptoms of stress; 90 percent had one or more symptoms to at least some degree. These symptoms included insomnia, nightmares, fearfulness, irritability and distressing recollections of the event. Although among the people surveyed, those who were closest to New York had the highest rate of stress reactions, others throughout the country, in large and small communities, also reported substantial stress reactions. They coped by talking with others (98 percent), turning to religion (90 percent), participating in-group activities (60 percent) and making donations (36 percent). Eighty-four percent of parents reported that they or other adults in the household had talked to their children about the attacks for an hour or more; 34 percent restricted their children television viewing. Thirty five percent of children had one or more stress symptoms, and 47 percent were worried about their own safety or the safety of loved ones.

In a nationally representative sample of Israel (Bleich et al., 2002) out of 512 participants, 84 (16.4 %) were directly exposed to the terrorist attack and 191 (37.3%) had a family member or a friend who had been exposed. 391 (76.7%) had at least one traumatic stress related symptom. Symptom criteria for PTSD were met by 48 (9.4 %) participants and criteria for acute stress disorder by one participant. 299 (58.6 %) reported feeling depressed. Female gender and use of tranquilizers, alcohol and cigarettes to cope were associated with TSR (traumatic stress related) symptoms and symptom criteria for PTSD.

In a recent survey of 2191 victims of terrorism in Northern Ireland (Curran and Miller, 2002) it was shown that 2

percent of the victims required admission and 13 percent were referred to either psychiatric outpatients, community psychiatric nurses or counselling services. Earlier studies such as by Lyons (1974) reported 4 percent psychiatric admission rates among the 100 individual victims of various bomb explosions in Northern Ireland. Curran (1988) reported that among the victims of Birmingham bombing, 5 percent required psychiatric help. Kee et al., (1987) observed that 4.5 percent of the surveyed 499 criminal injury litigants who had each been victim of a variety of acts of terrorist and criminal violence, required psychiatric in patient admission while 11 percent reported to NHS out patient services. Out of these 499 victims, 23 percent had a diagnosis of PTSD.

In their study on the 182 adult survivors of the bombing at a federal building at Oklahoma, North et al. (1999) reported that 4.5 percent of the subjects had a post disaster disorder. Out of these 34.6 percent had PTSD followed by major depressive disorder (22%) and panic disorder (6.6 %). Predictors of the impact included disaster exposure, female gender, and pre-disaster psychopathology. Onset of the PTSD was swift with 76% reporting the same day onset.

As already mentioned, the published Indian data in this area is dismally minimal. In their study, Margoob et al. (2001) reported significant increase in the number of individuals seeking treatment at a general hospital psychiatric unit (GHPU) in Srinagar. Their number has risen from a total of 1762 in 1990 (when terrorism in Kashmir was just germinating) to 37860 in the year 2001. This marked increase of attendance in a psychiatric OPD cannot be possibly explained by any other factor except the growing impact of terrorism and violence. In the year 2001 a significant number of patients (2.38 %) were diagnosed to be suffering from PTSD. Among these 68.2 percent had immediate onset and 31.8 percent had delayed onset i.e. onset after 6 months of the traumatic event.

Gautam et al (1998) in their study on the victims (n=31) of a bomb blast in a bus caused by terrorist activity reported 35.4 percent of psychiatric morbidity at day 3 and 29.3 percent after 2 weeks. After 2 weeks the most common ICD.10 psychiatric diagnosis was PTSD (12.9 %) followed by depression (9.6 %) and dissociative amnesia (6.4 %).

In the light of the available literature it cannot be denied that disaster in the form of terrorism leads to significant mental disequilibrium and psychiatric morbidity .It definitely represents a major challenge with regard to designing an effective strategy for coping with the aftermath of such an attack.

### Impact on counterinsurgents

While talking about the psychological impact of terrorism an important group cannot be left untouched i.e. the troops, which are involved, in counter insurgency operations. The Indian Army has been engaged in counter insurgency operations from time to time since 1948. The level and complexity of such operations has risen steeply since 1984. A new and more dangerous dimension was added in 1989 when Pakistan stepped up its proxy war in Jammu and Kashmir (Prasad, 1992). While the nation has been technically at peace, the army has been at war and has suffered more casualties in such 'low intensity' operations than in the three major conflicts with Pakistan. Soldiering in a 'low intensity' conflict scenario involves significant psychological stress due to its chronic and incessant nature and ever-present threatening situation. The problem of combat related stress has received considerable attention in the West particularly in United States (Driskell, 1991; Sutker, 1993). Very few Indian researchers have worked upon the psychological issues involved in such counter insurgency operations (Prasad, 1992, Grewal and Khanna, 1992; Raza, 1992; Sardeshpande, 1992; Kharab, 1992; Goel, 1997; Goel, 1998). Goel (1998) reported that the most important operational factor affecting the morale of army officers involved in counter insurgency operations were their bitterness at the inability to deal with "Jamayatis" (Individuals blatantly misusing religious institutions in their antinational activities) followed by the 'anger at fighting with constraints'. The reverse was observed among the personnel other than officers.

### Impact on Children

Terrorism adversely affects the psyche of the children who are directly or indirectly exposed to it. They may have been the direct victim or witness of the violence or they suffer because of the loss or disability of their parents and caregivers. Children react differently to traumatic events depending on their age. Younger children may show abnormal behaviour in the form of persistent fear of being separated, excessive clinging, crying, screaming, sleep problems or develop nightmares and regressive behaviour. Older children, may become withdrawn from others / activities, show disruptive behaviour, are unable to concentrate, become fearful and irritable, develop irrational anger and fear, become depressed or anxious and achieve lower grades. Adolescents may develop symptoms of PTSD, abuse drugs and alcohol and may develop suicidal thoughts. ' Traumatic play' a specific form of re-experiencing seen in children, consists of repetitive acting out of the trauma or trauma related themes in play. In their study Jones et al (2003) reported on referring problems and psychiatric diagnoses in 559 children attending a locally established child and adolescent psychiatric service over two years in Kosovo, Yugoslavia, in the immediate aftermath of the NATO air strikes. They found that non-organic enuresis; behavioural problems, fear and learning difficulties were the most common problems. Considering the significant impact on children there is a need to develop a sustainable, community based child and adolescent mental health service that attempts to address full range of mental health problems. By doing so we could prevent the development of maladaptive behaviour patterns in the affected child and adolescent population.

### How can we help ?

Any human made disaster leaves people with a great sense of betrayal, ripping apart the social fabric that is essential for any person's sense of well being. In order to take care of the emotional needs of the trauma affected people few major challenges need to be considered are:

1. Severe stress and trauma due to violence
2. Sudden forced displacement.
3. Uncertainty about the future and the continuation of threat.
4. Process of rebuilding personal, family and community life.

Chronic and large-scale violence in any form exerts its pressure on the mental health care manpower and infrastructure. Both the attendance in psychiatric OPD and psychiatric admissions increase considerably. This increased burden can only be tackled by enhancing the number of mental health care delivery personnel including psychiatrist, clinical psychologist, psychiatric nurses and psychiatric social workers and other paramedics. It is not anticipated that the mental health services would be among the ranks of frontline responders however they should play a signal role in developing an effective multi-disciplinary response, particularly with regard to the reduction of public anxiety, identifying at risk individuals, providing crisis intervention and collaborating with medical and emergency services, as well as offering care for those who develop post-traumatic psychopathology. There is a need to offer an empathic, non-judgemental, collaborative approach to help these ailing individuals to achieve a better level of adjustment. According to DiGiovanni, (1999) there are a number of key roles that the mental health professionals could be expected to fulfill: advising the authorities on how to manage anxious and distressed individuals; providing advice for surgical and medical staff about post-traumatic reactions; helping to determine that symptoms such as tachycardia, tension, nausea and tremor could be psychological reactions to stress and conducting triage to identify those in need of more specialist psychiatric care. In developing countries like ours, the resources devoted to mental health are often inadequate to meet even routine needs. The primary health care system is an important network available .For the affected population the assistance should be directed at mobilizing local strengths wherever possible. Community level interventions are important to address the major challenges in the aftermath of terrorism. Simple community interventions are provided first .For those individuals with particular medical and specific needs, specialist care is made available later. In other words the psychiatric / psychological interventions are not offered indiscriminately. Beyond the clinical inputs from the psychiatrists, their skills as a team leader are warranted in such situations. Guiding and training volunteers, social workers and NGO's would ultimately result in the better care of the victims of trauma and violence. Recognition of community participation and the support of its members is the foundation for a speedy reconstruction and rehabilitation process. An outshining example of such an effort is the creation of a cadre of bare foot mental health counselors by Rajiv Gandhi Foundation in Budgam district of J & K which is intended to ease suffering and depression of people affected by militancy (Hindustan Times, 2003). An indirect relevance can be drawn from the initiatives in Indian settings in the post disaster mental health care provided to the sufferers by various governmental bodies and NGO's in: Bhopal after gas tragedy, Gujarat after earthquake and riots, Mumbai after riots, Latur after earthquake and post super cyclone Orissa. As an after thought, I would like to add that " terrorism breeds terrorism" and thus by healing the mentally injured victims we can prevent them to become propagators of such hideous activities.

### Conclusion

Psychological sequelae are seen commonly after any form of mass violence. Any act of terrorism by the nature of its very purpose leaves a lingering impact on those who are either its victim or even its witness. Several of the surveys and studies worldwide have confirmed this observation. In the current trend of increasing global and national terrorist and violent activities, it is being actively discussed about the possible role, which we, as mental health professionals can play. This however cannot be denied that most of the interventions after any form of disaster would be preventive in nature and can be done by volunteers after preliminary orientation training. The contribution of psychiatrists can be both in the form of a clinician for treating individuals with morbid post terrorism psychopathology and also educational i.e. educating the volunteers, NGOs and other professionals about the nature of psychiatric manifestation and their management at the community level. I would like to end my address on a positive note. The natural resilience of the individual and communities should never be underestimated. There can also be some positive gains following any catastrophe, including: a more united community; individuals identifying new strengths; relationships becoming more closely bonded; and life priorities and values being constructively revised.

### ACKNOWLEDGEMENT

I would like to acknowledge the contributions in the preparation of my address by Dr. Kartikey Agarwal (Resident, Department of Psychiatry, K.G.M.University), Dr.Rajul Tandon(Research Associate, K.G.M.University, Lucknow), Prof. R.S.Murthy, Prof. Mushtaq Margoob,Prof.D.S.Goel,Prof.R.L.Kapoor,colleagues in the department of psychiatry and all the members of IPS.

### REFERENCES

Blaney, P.H. (1986) Affect and memory: a review. Psychological Review, 99,229-246.

Blazer, D.G., Kessler,R.C., McGonagle, K.A., Swartz,M.S.(1994) The prevalence and distribution of major depression in a national community sample: the National Comorbidity Survey. American Journal of Psychiatry, 151:979-986.

Bleich, A.,Gelkopf,M.,Solomon,Z. (2002) Exposure to terrorism, stress - related mental health symptoms, and coping behaviours among a nationally representative sample in Israel.

Crenshaw, M.(1990) The logic of terrorism: terrorist behaviour as a product of strategic choice. In Origins of Terrorism. Cambridge: Cambridge University press.

Curran, P.S. & Miller ,P.W ( 2002) Psychiatric implications of chronic civilian strife or war: Northern Ireland.

Curran,P.S.(1988) Psychiatric aspects of terrorist violence: Northern Ireland, 1969-1987. British Journal of Psychiatry, 153,470-475.

Department of Health and Human Services. Mental health (1999) A report of the Surgeon General. Rockville,Md.: Substance Abuse and Mental Health Services Adminstration, Center for Mental Health Services, National Institute of Mental Health Services.

DiGiovanni,C.(1999) domestic terrorism with chemical or biological agents: psychiatric aspects. American Journal of Psychiatry,10,1500-1505.

Doty,R.M., Peterson,B.E. & Winter,D.G.(1991) Threat and authoritarianism in the United States, 1978-1987.Journal of Personality and Social Psychology,61,629-640.

Driskell,J. (1991) Effects of stress on military performance in : Handbook of military psychology, Gal R & Mangelsdorf AD,(Eds.), John Wiley, New York, 84-88.

Engel,C.C.(2001) Outbreaks of medically unexplained physical symptoms after military action, terrorist threat or technological disaster, Military Medicine,166 (suppl.,2), 47-48.

Ferraro,K.A.(1996) Women's fear of victimization: shadow of sexual assault ? Social Forces, 75,667-690.

Figley, C.R.& Stretch,R.H. (1980) Vietnam Veterans Questionnaire Combat Exposure Scale. In: Figley, Stretch,R.H. (Eds.) Vietnam Veterans Questionnaire. Instrument development. West Lafayette, In: Family Research Institute, Purdue University.

Galea, S. , Ahern,J., Resnick,H., Kilpatrick,D., Bucuvalas,M., Gold,J., Vlahov,D. et al.(2002) Psychological sequelae of the September, 11, Terrorist Attacks in New York City. The New England Journal of Medicine, Vol.346, No.13, March 28,2002.

Gautam, S., Gupta, I.D., Batra, L., Sharma, H., Khandelwal,R. & Pant, A. ( 1998) Psychiatric morbidity among victims of bomb blast. Indian Journal of Psychiatry, 40(1) 41-45.

Gilligan,S.G. & Bower, G.H.(1984) Cognitive consequences of emotional arousal. In C. Izard., J.Kagan, & R.Zajonc (Eds.), Emotions, Cognition, and Behavour . New York:Cambridge.

Goel,D.S.(1997) Low intensity conflicts: psychological aspects. Paper presented at CME (Military Psychiatry), Kiekee Sep.

Goel,D.S.(1998) Psychological Aspects of Counter Insurgency Operations. Combat Journal, 27(1): 43-48.

Goenjian, A.K., Molina,L., Steinberg,A.M., et al. (2001) Posttraumatic stress and depressive reactions among Nicaraguan adolescents after hurricane Mitch. American Journal of Psychiatry,158:788-794.

Greenberg,J.,Simon,L., Pyszczynski,T., Solomon,S. & Chatel,D.(1992) Terror management and tolerance: does mortality salience always intensify negative reactions to others who threaten one's world view ? Journal of Personality and Social Psychology, 63,212-220.

Grewal,S.S.& Khanna,B.K. (1992) Low intensity conflict in the Indian context, USI Journal,72:205-223.

Grinker,K.& Spiegel,S.(1945) Men under stress. Philadelphia, Pa:Lewiston.

Hindustan Times (Dec, 22-03) New Delhi.

Huddy, L., Feldman, S., Taber,C. & Lahav,G.(2002) The psychological consequences of perceived threat. Unpublished manuscript. SUNY at Stony Brook.

Jacobson, D. & Bar-Tal, D. (1995) Structure of security beliefs among Israeli students. Political Psychology, 16,567-590.

Jones,F. & Fong,Y.(1994) Military psychiatry and terrorism. In Department of the Army, Textbook of military medicine (pp.264-269). Washington, D.C.: Department of the Army.

Jones,L., Rrustemi,A.,Shahini,M.,Uka,A.. (2003) Mental Health services for war-affected children, Report of a survey in Kosovo, British Journal of Psychiatry, 183,540-546.

Kahneman,D.& Tversky,A.(1979) Prospect theory: an analysis of decisions under risk. Econometrica, 47,263-291.

Kaplan,A.(1981) The psychodynamics of terrorism. In Y.Alexander & J.Gleason (Eds.)Behavioural and quantitative perspectives on terrorism (pp.35-50). New York. Pergamon.

Kapur,R.L.(1994) Violence in India: A Psychological Perspective . D.L.N.Murthy Rao Oration, Indian Journal of Psychiatry, 36(4), 163-169.

Kar,G.C.(2000) Disaster and Mental Health . Presidential address: Delivered at Annual National Conference of Indian Psychiatric Society at Cochin.

Kee,M., Bell,P., Loughrey,G.C., et al. (1987) Victims of violence: a demographic and clinical study. Medicine, Science and the Law, 27,241-247.

Kharb,K.S. (1992) Joint civil military response to terrorism. Combat Journal,19(2) :14-22.

LeVine,R.A. & Campbell,D.T.(1972) Ethnocentrism: theories of conflict, ethnic attitudes and behaviour. New York: John Wiley.

Liberman,A. & Chaiken,S.(1993) Defensive processing of personally relevant health messages. Personality and Social Psychology Bulletin.

Lodge,M. & Taber,C.(2000) Three steps toward a theory of motivated political reasoning. In: A.Lupia, M.McCubbins & S.Popkin (Eds.), Elements of political reasoning: understanding and expanding the limits of rationality.London:Cambridge University press

Lyons, H.A. (1974)Terrorist bombing and the psychological sequelae. Journal of the Irish Medical Association,67,15-19.

Marcus, G.E., Sullivan,J.L. , Theiss-Morse,E. & Wood,S.L.(1995) With malice toward some: how people make civil liberties judgments. Cambridge ,MA: Cambridge University Press.

Margoob et al. (2001) Personnel Communication.

Mathews,A. & MacLeod,C.(1986) Discrimination of threat cues without awareness in anxiety states. Journal of Abnormal Psychology, 95: 131-138.

Myers, D.(1994) Disaster response and Recovery: A hand book for Mental Health Professionals. US Department of Health and Human Services.

North ,C.S., Nixon,S.J., Shariat,S. et al.(1999) Psychiatric disorders among survivors of the Oklahoma City bombing, JAMA, 282: 755-62.

North,C.S., Nixon ,S.J., Shariat,S. et al. (1999) Psychiatric disorders among survivors of the Oklahoma City bombing.JAMA, 282: 755-62.

Post, J.M.(1990) Terrorist Psycho-logic: Terrorist behaviour as a product of psychological forces. In Origins of Terrorism. Cambridge: Cambridge University Press.

Prasad,B.A.(1992) Holocaust in Kashmir: a review, Combat Journal ,19,10-20.

Prevention of Terrorism Act (2002) Second edition, Eastern Book Company,Lucknow.

Raza, M. (1992) Beyond guerilla warfare: a new dimension. Combat Journal, 19(2),3-13.

Rookeach,M.(1960) The open and closed mind. New York: Basic Books.

Sardeshpande, S.C. (1992) Military and national security. Combat Journal, 19(4), 3-9.

Schuster,M.A., Bradely,D.S.,Jaycox,J.HL, Collins,L.R., Marshall,G.N., Elliott,N.M., Zhou, J.A., Kanouse,D.E., Janina,L. Morrison,A.B. & Berry,H.B.(2001) A national survey of stress reactions after the September,11,2001 ,terrorist attacks N.Engl.J.Med., Vol., 345, No.2.

Shore,J.H., Vollmer,W.M., Tatum,E.L. (1989) Community patterns of postrraumatic stress disorders. J.Nerv.Ment.Dis.,177: 681-5.

Singh,R.A. & Singh,A.S.( 2003) Psychiatric Consequences of WTC Collapse and the Gulf war.Mens Sana Monograph ,Mens Sana Research Foundation .Mumbai,India.

Sprinzak,E.(1990) The psychological formation of extreme left terrorism in democracy: the case of the weathermen. In Origins of Terrorism. Cambridge: Cambridge University Press.

Struch,N. & Schwartz,H.S.(1989) Intergroup aggression: its predictors and distinctiveness from in group bias. Journal of Personality and Social Psychology,56,364-373.

Sutker,P.B. (1993) War-zone trauma and stress related symptoms in Operation Desert Shield/ Storm (ODS) returnees. Journal of Social Issue, 49,33-49.

Taylor,M.(1988) The terrorist. London: Brassey’s Defense

Tyhurst,J.S.(1951) Individual reactions to community disaster: the natural history of psychiatric phenomena. American Journal of Psychiatry,107,764-769.

Department of state(2000) Patterns of global terrorism,(online). Available: http// www.governmentguide.com/govsite.adp.

